# Water-Soluble Fish Protein Intake Led to Lower Serum and Liver Cholesterol Concentrations in Obese Zucker fa/fa Rats

**DOI:** 10.3390/md16050149

**Published:** 2018-05-01

**Authors:** Aslaug Drotningsvik, Linn Anja Vikøren, Svein Are Mjøs, Åge Oterhals, Daniela Pampanin, Ola Flesland, Oddrun Anita Gudbrandsen

**Affiliations:** 1 Dietary Protein Research Group, Department of Clinical Medicine, University of Bergen, 5021 Bergen, Norway; aslaug.drotningsvik@uib.no (A.D.); linn.vikoren@uib.no (L.A.V.); 2 Vedde AS, TripleNine Group, 6030 Langevåg, Norway; ola.flesland@vedde.no; 3 Department of Clinical Science, University of Bergen, 5021 Bergen, Norway; 4 Department of Chemistry, University of Bergen, P.O. Box 7803, 5020 Bergen, Norway; Svein.Mjos@uib.no; 5 Nofima AS, P.O. Box 1425 Oasen, 5828 Bergen, Norway; Aage.Oterhals@nofima.no; 6 International Research Institute of Stavanger, Mekjarvik 12, 4070 Randaberg, Norway; Daniela.Pampanin@iris.no

**Keywords:** cardiovascular disease, fish protein, cholesterol, hydroxymethylglutaryl-CoA reductases, LDL receptor, Zucker rats

## Abstract

Proteins from different fish species and different raw materials such as fish fillets and by-products have shown promising cardioprotective effects in rodents and humans, including effects on cholesterol metabolism. Blue whiting is used mainly to produce fish meal for the feed industry and during this production, a water-soluble protein fraction, containing small peptides that are easily absorbed and may hold bioactive properties, is isolated. The effects of water-soluble fish protein on cholesterol metabolism were investigated in twelve male obese Zucker fa/fa rats. Rats were fed diets with water-soluble protein from blue whiting (BWW) as 1/3 of the total protein and the remaining 2/3 as casein (BWW group) or with casein as the sole protein source (control group). After 5 weeks intervention, the BWW group had lower serum total, high-density lipoprotein (HDL), and low-density lipoprotein (LDL) cholesterol concentrations and lower cholesteryl ester concentration compared to controls. Hepatic concentrations of cholesterol, 3-hydroxy-3-methylglutaryl coenzyme A (HMG-CoA) reductase, and LDL receptors were also lower in the BWW group. The groups had a similar concentration of serum total bile acids and similar fecal excretions of cholesterol and bile acids. To conclude, the BWW diet led to lower concentrations of serum and liver cholesterol in obese Zucker fa/fa rats, probably due to lower hepatic cholesterol synthesis.

## 1. Introduction

Cardiovascular disease is one of the leading causes of death worldwide [1] and is associated with risk factors such as obesity and dyslipidemia [2]. Primary prevention strategies for cardiovascular disease focus on reducing risk factors by lifestyle modifications [3], and include recommendations from the American Heart Association and the British National Health Service to consume at least two weekly servings of fish [4,5]. Fish consumption is associated with reduced risk of coronary heart disease [6,7] and this effect is thought to be partly explained by the high content of long-chain n-3 polyunsaturated fatty acids that are found especially in fatty fish [8]. Recently, intake of fish protein has been shown to lower circulating cholesterol in overweight humans [9] and rodents [10,11,12,13,14,15,16,17], indicating that not only fish oil but also fish protein may affect risk factors for cardiovascular disease. 

Blue whiting (*Micromesistius poutassou*) is primarily used to produce fish meal for the aquaculture industry and is utilized only to a limited extent for human consumption [18]. Proteins from blue whiting may be upgraded to products suitable for human consumption, thereby leading to products with higher value. Fish meal can be produced from either the whole fish or fish by-products such as heads, guts, and bones. During this production process, the aqueous fraction, containing water-soluble protein (stickwater), is separated from the solid phase of the fish material [19]. Small peptides, free amino acids, and low molecular weight compounds such as taurine are found in the water-soluble fish protein fraction [20,21]. Unlike large proteins, short peptides (mainly dipeptides and tripeptides) can produce local effects in the digestive tract or enter the circulatory system without prior digestion and thus exert effects as bioactive compounds [22]. Documenting the effects of water-soluble proteins from blue whiting could lead to improved utilization of blue whiting proteins and promote the development of dietary supplements targeting risk factors for cardiovascular disease. 

The obese Zucker fa/fa rat is the most widely used rat model for studies of metabolic complications and for possible treatments of obesity in humans [23]. The Zucker fa/fa rat develops obesity due to a defect in the leptin receptor [24] and presents visible obesity already at four weeks of age [25]. Metabolic abnormalities in these rats include elevated concentrations of serum triacylglycerols, and low-density lipoprotein (LDL), high-density lipoprotein (HDL), and very low-density lipoprotein (VLDL) cholesterol [26]. 

The main aim of the present study was to investigate the effects of a diet containing water-soluble protein from blue whiting (BWW) on cholesterol metabolism in obese Zucker fa/fa rats. We hypothesized that feeding obese Zucker fa/fa rats a BWW diet would lead to a lower concentration of serum cholesterol compared to rats fed casein as the sole protein source. To investigate whether a BWW diet affects cholesterol metabolism, we analyzed serum cholesterol and bile acids, fecal excretion of cholesterol and bile acids, as well as concentrations of cholesterol, LDL receptors, and 3-hydroxy-3-methylglutaryl coenzyme A (HMG-CoA) reductase in liver. In line with our hypothesis, we found that feeding obese Zucker fa/fa rats a diet containing water-soluble fish protein led to a lower serum cholesterol concentration. 

## 2. Results

### 2.1. Dietary Compositions

The amino acid composition differed between the two diets, with a slightly lower content of all indispensable amino acids in the BWW diet compared to the control diet. The glycine content was higher and the ratios of lysine/arginine and methionine/glycine were lower in the BWW diet than in the control diet, whereas taurine was detected only in the BWW diet (Table 1). The fatty acid composition was similar between the diets, except for trace amounts of 20:5n-3 and 22:6n-3 in the BWW diet (Table 1). The dietary cholesterol content was similar between the BWW diet and control diet with 0.23 and 0.24 µmol cholesterol per gram diet, respectively. The water-soluble fish protein used in the BWW diet consisted of 36.6% peptides with molecular weights larger than 20,000 g/mol, 12.7% peptides with molecular weights in the range of 10,000–20,000 g/mol, 8% peptides with molecular weights in the range of 500–10,000 g/mol, and 5.5% peptides with molecular weights between 200 g/mol and 500 g/mol, while the peptide fraction smaller than 200 g/mol (comprising free amino acids and miscellaneous water-soluble components absorbing light with a wavelength of 214 nm) amounted to 37.3%. We searched for the hypocholesterolemic motifs ALPMH, GGV, GLDIQK, HIRL, IAVPGEVA, IIAEK, LPYPR, PGPL, VAWWMY, VGVI, VGVL, VPDPR, and VYVEELKPTPEGDLEILLQK in the water-soluble protein from blue whiting and of these only GGV was identified.

### 2.2. Growth and Energy Intake

Rats in the BWW group had significantly lower body weight at baseline when compared to the control group (Table 2). Growth during the intervention period, the 24 h energy intake, as well as body weight to square body length ratio and body weight at time of euthanasia were similar between the groups.

### 2.3. Cholesterol and Bile Acids in Serum

Serum concentrations of total cholesterol, cholesteryl ester, LDL cholesterol and HDL cholesterol were significantly lower in the BWW group compared to the control group, while the serum concentration of total bile acids was similar between the two groups (Table 3).

### 2.4. Cholesterol, HMG-CoA Reductase, and LDL Receptor Concentrations in Liver and Fecal Cholesterol and Bile Acids

**Figure 1:**
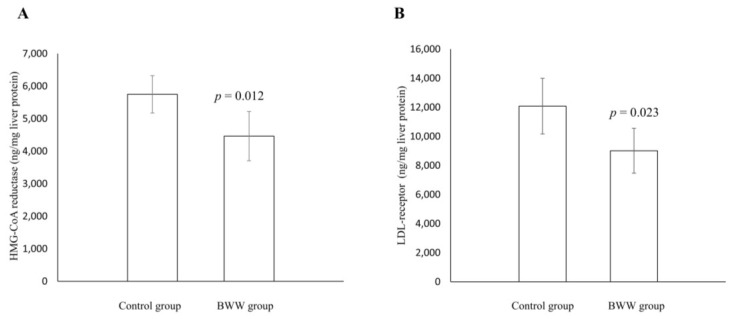
Effects of the BWW diet compared to the control diet on liver concentrations of HMG-CoA reductase (**A**) and LDL receptors (**B**) shown relative to protein. Values are presented as the mean with their standard deviation shown by vertical bars for *N* = 5 rats in the control group and *N* = 6 rats in the BWW group. *p* < 0.05 was considered significant; evaluated by independent samples *t*-test; BWW: blue whiting water-soluble protein; HMG-CoA: 3-hydroxy-3-methylglutaryl coenzyme A; LDL: low-density lipoprotein.

The concentration of liver cholesterol was significantly lower in the BWW group compared to the control group (Table 4). Concomitant with this, the BWW group had significantly lower liver concentrations of HMG-CoA reductase and LDL receptors compared to the control group (Figure 1A,B). Fecal daily output of total cholesterol and bile acids were similar between the two groups (Table 4). 

## 3. Discussion

Cardiovascular disease is one of the leading causes of death worldwide and elevated circulating cholesterol is considered an important risk factor for the development of this disease [2]. Some fish proteins have shown promising effects on cholesterol metabolism in obese Zucker fa/fa rats but the effects seem to differ among proteins from various fish species, such as herring, salmon, and cod, and among protein fractions with different peptide sizes [12,15,27,28]. In the present study, we show that feeding obese Zucker fa/fa rats a diet with water-soluble protein from blue whiting as 1/3 of the total dietary protein content leads to lower serum and liver cholesterol concentrations. Concomitant with this, we found lower liver concentrations of LDL receptors and HMG-CoA reductase in the BWW group compared to the control rats fed diets with casein as the sole protein source. The lower serum cholesterol concentration accompanied with a lower liver HMG-CoA reductase concentration is of immense interest since the primary target for medical treatment of high circulating cholesterol is a reduction in liver HMG-CoA reductase activity. Our results indicate that water-soluble protein from blue whiting holds hypocholesterolemic properties and could act as a functional ingredient by reducing risk factors for cardiovascular disease. To our knowledge, this is the first study to investigate the effects of water-soluble protein from blue whiting on cholesterol metabolism in obese Zucker fa/fa rats.

Lower concentrations of circulating total cholesterol and LDL cholesterol are associated with reduced risk of cardiovascular disease [29] and it is, therefore, of interest to identify nutrients with cholesterol-lowering capacities that may contribute to cardiovascular disease prevention. Rats fed a BWW diet had lower concentrations of serum total cholesterol, cholesteryl esters, LDL cholesterol and HDL cholesterol and a lower concentration of liver cholesterol when compared to control rats. We have previously found lower serum LDL and HDL cholesterol concentrations in obese Zucker fa/fa rats fed diets with 25% of total dietary protein from rest raw material of herring hydrolysate [15] or 100% of total dietary protein from salmon hydrolysate [12]. In contrast to this, no effects on serum cholesterol concentrations were seen after intake of a diet with 25% of total protein from salmon hydrolysate from rest raw material [15] or 25% of total protein from cod protein [27] in obese Zucker fa/fa rats. These diverging results underline the need for further research on the effects of different fish proteins and peptides on cholesterol metabolism.

Cholesterol concentrations in circulation and in the liver are affected by intake, synthesis, uptake by the liver and extrahepatic tissues, as well as fecal excretion of cholesterol and bile acids. HMG-CoA reductase is the rate limiting enzyme in cholesterol synthesis, and inhibition of this enzyme is the primary target for the medical treatment of hypercholesterolemia [30]. The lower serum and liver cholesterol concentrations in the BWW group compared to the control group may be caused by a lower liver cholesterol biosynthesis since the hepatic concentration of HMG-CoA reductase was lower in the BWW group. High cholesterol intake can lead to a downregulation of liver HMG-CoA reductase activity, but in the present study the dietary cholesterol content was similar between diets. The bioactive motif GGV was identified in the water-soluble protein from blue whiting and this is of interest in this setting since GGV has been reported to be a strong inhibitor of HMG-CoA reductase activity in vitro [31]. Therefore, the presence of GGV in the BWW diet may have contributed to the lower hepatic HMG-CoA reductase concentration in the present study and, thereby, to the lower cholesterol concentrations in the liver and consequently in serum. Moreover, the hepatic LDL receptor concentration was lower in the BWW group compared to the control group, implying that a lower uptake of LDL cholesterol from the circulation may have contributed to the lower liver cholesterol concentration in the BWW-fed rats. It is tempting to speculate that the lower LDL receptor concentration in the liver is a consequence of the lower LDL cholesterol concentration in the serum. Another route for clearance of cholesterol from the circulation is via the scavenger receptor class B type I, which mediates the transfer of cholesteryl esters from HDL cholesterol to the liver where cholesterol can be further metabolized and secreted in the bile [32]. Since the BWW group had a lower liver cholesterol concentration combined with similar fecal excretion of cholesterol and bile acids when compared to the control rats, it is not likely that the lower serum cholesterol in the BWW rats is a consequence of increased cholesterol uptake via the liver scavenger receptor class B type I. Based on the findings that the BWW rats had a lower concentration of HMG-CoA reductase and LDL receptors in liver, it is more plausible that the lower amounts of circulating and liver cholesterol is mediated through a downregulation of hepatic cholesterol synthesis. Sterol regulatory element-binding protein (SREPB)-2 also plays an important role in the regulation of liver cholesterol metabolism, including the regulation of liver HMG-CoA reductase and LDL receptors [33]. It is, therefore, of interest that fish protein feeding has also been reported to result in both higher liver cholesterol concentrations and upregulated gene expressions of HMG-CoA reductase, LDL receptors, and SREBP-2 in rats [16]. Thus, fish proteins may affect hepatic genes involved in cholesterol metabolism and it is possible that the lower liver HMG-CoA reductase and LDL receptor concentrations in the BWW group in the present study is mediated through a downregulation of the SREBP-2 in liver.

The major route of cholesterol removal from the body is through the excretion of bile acids, and lower circulating cholesterol concentrations after fish protein feeding have been explained by increased fecal excretion of cholesterol and/or bile acids [10,17]. Rats conjugate the bile acid cholic acid with both glycine and taurine, but prefer taurine [34], and a cholesterol lowering effect of taurine has been suggested [35]. Taurine was found in low amounts in the BWW diet, but not in the control diet, and may have contributed to the lower serum and liver cholesterol concentrations in the BWW group by conjugating bile acids for fecal excretion. However, as the fecal excretion of both bile acids and cholesterol were similar and no difference was observed in serum bile acid concentration between the groups, this probably does not explain the lower serum and liver cholesterol concentrations in the BWW group compared to the control rats. 

The present study has some limitations. Rats in the BWW group had a 4.7% lower baseline weight compared to the control group and this difference in body weight could have affected the results. However, the energy intake, growth, endpoint weight, and body weight to square body length ratio were similar between the groups, suggesting that the lower baseline body weight in the BWW group did not contribute to the observed differences in cholesterol metabolism between the two groups. To investigate the mechanisms behind the hypocholesterolemic effects of the BWW diet, we measured the concentrations of HMG-CoA reductase and LDL receptors in liver. Enzyme activities were not measured since such analyses should be conducted in fresh liver samples and, therefore, we cannot conclude whether the BWW diet affects hepatic activities of HMG-CoA reductase and LDL receptors. The difference in cholesterol transport between humans and rats should be considered when results from the present study are discussed in relation to human cholesterol metabolism. Obese Zucker fa/fa rats carry most of the circulating cholesterol in the HDL particle, in contrast to humans where circulating cholesterol is mainly carried in the LDL particle [23,36]. Also, the development of obesity in the Zucker fa/fa rat is caused by a genetic defect in the leptin receptor that is rare in humans. Therefore, the effects of blue whiting water-soluble proteins on cholesterol metabolism should be further investigated in humans.

## 4. Materials and Methods

### 4.1. Ethical Statement

The study protocol was approved by the National Animal Research Authority (Norway) in accordance with the Animal Welfare Act and the Regulation of Animal Experiments (approval number 2014/6979). All applicable international, national, and institutional guidelines for the care and use of research animals were followed.

### 4.2. Animals and Diets

Twelve male, obese Zucker fa/fa rats (HsdHlr:ZUCKER-Lepr^fa^) were obtained from Harlan Laboratories (Indianapolis, IN, USA). The rats were housed in pairs based on weight in Macrolon IV cages in a room with a 12 h light/dark cycle, at 20–23 °C, and a relative humidity of 55–65%. Rats were acclimatized for at least 7 days under these conditions before being allocated to either intervention group blue whiting water-soluble protein (BWW) or control group. Rats were fed experimental diets based on the American Institute of Nutrition’s recommendation for growing laboratory rodents (AIN-93G) [37] that differed only in their protein sources. Both diets contained 20 wt % protein; the BWW diet consisted of 1/3 of total protein from blue whiting water-soluble protein and 2/3 of protein from casein. In the control diet, casein was the sole protein source (Table 5). All ingredients were purchased from Dyets Inc. (Bethlehem, PA, USA) except casein, which was purchased from Sigma-Aldrich (Munich, Germany), and the water-soluble protein, which was prepared from blue whiting (*Micromesistius poutassou*) by Nofima (Bergen, Norway). 

### 4.3. Preparation of Blue Whiting Water-Soluble Protein

The blue whiting was frozen onboard the fishing vessel, landed, and then partially thawed at ambient temperature, headed, and gutted before it was refrozen. The frozen raw material was partially thawed overnight, added an equal amount of water by weight and heated to 90 °C. After 10 min holding time, the cooked material was mechanically dewatered in a P13-SCR double-screw press (Stord Bartz AS, Bergen, Norway) and the press liquid was collected. To the obtained press cake, an equal amount of water was added. It was then heated and pressed once more at the same conditions to extract residual water-soluble compounds. The press liquids were pooled, heated to 90 °C, and run through a Jesma VS 20/65 Roto-Fluid sieve (Jesma, Velje, Denmark; 100 μm sieve net opening) to remove suspended solids before concentrating in a four-stage falling film evaporator (APV Anhydro, Soeborg, Denmark) at 60–100 °C. The concentrate was centrifuged in a Sorvall RC5C centrifuge (13,200× *g*, 10 min, Sorvall Instruments, Wilmington, DE, USA) to remove fine suspended particles before freeze-drying and final milling on a Retsch ZM-1 centrifugal mill (Retsch GmbH, Haan, Germany) with a ring sieve aperture of 1.0 mm. 

### 4.4. Design 

Rats were fed ad libitum for five weeks with free access to tap water and chewing sticks. The intervention period started when the rats weighed between 290 g and 325 g. Rats were weighed weekly during the intervention period. One week before the endpoint, rats were housed individually in metabolic cages (Ancare Corp., New York, NY, USA) for 24 h for collection of feces and measurement of feed intake, without fasting in advance. At the end of the intervention period, after a 12 h fast with free access to tap water, rats were euthanized with Isofluran (Isoba vet, Intervet, Schering-Plough Animal Health, Boxmeer, The Netherlands) mixed with nitrous oxide and oxygen. Rats were euthanized during the morning in a randomized order. Blood was drawn directly from the heart and collected in Vacuette Z Serum Clot Activator Tubes (Greiner Bio-one, Kremsmünster, Austria) for isolation of serum. Liver was dissected out and frozen. Serum, liver, and fecal samples were stored at −80 °C until analysis. Body length (without tail) was measured using a ruler.

### 4.5. Analyses of Diets

Peptide size distribution of the water-soluble blue whiting protein, and amino acid composition and energy content in the diets were analyzed by Nofima BioLab (Bergen, Norway). The lipids in the diets were extracted according to the method described by Bligh and Dyer [38] using a mixture of methanol and chloroform. The fatty acid composition of the diets were analyzed by GC after lipid extraction and methylation, as described previously [27,38]. Dietary lipid extracts were evaporated to dryness using nitrogen and re-dissolved in isopropanol before quantification of total cholesterol on the Cobas c111 system (Roche Diagnostics GmbH, Marburg, Germany) using the CHOL2 (Cholesterol Gen.2) kit from Roche Diagnostics. The identification of bioactive motifs in the water-soluble blue whiting protein was performed by the International Research Institute of Stavanger (IRIS, Stavanger, Norway) as described by Pampanin et al. [39], using LC-MS Orbitrap analyses (LC-MS/MS) with the Sequest algorithm to search for the following peptide sequences, which have been reported to have cholesterol-lowering properties: ALPMH [40], GGV [31], GLDIQK [40], HIRL [41], IAVPGEVA [42], IIAEK [40], LPYPR [43], PGPL [44], VAWWMY [45], VGVI [31], VGVL [31], VPDPR [46], and VYVEELKPTPEGDLEILLQK [40]. Peptide sequences identified in the water-soluble blue whiting protein are presented in the Supplementary Material.

### 4.6. Serum Analyses

Serum total and LDL cholesterol were quantified at the Laboratory of Clinical Biochemistry at Haukeland University Hospital (Bergen, Norway) by accredited methods. Serum HDL cholesterol (4× dilution with saline), free cholesterol, and total bile acids were analyzed on the Cobas c111 system using the HDLC3 (HDL cholesterol) kit from Roche Diagnostics, Free Cholesterol FS kit (Diagnostic Systems GmbH, Holzheim, Germany), and Total Bile Acid Assay kit (Diazyme Laboratories, Inc., Poway, CA, USA). Cholesteryl ester concentration was calculated as the difference between total and free cholesterol. 

### 4.7. Cholesterol in Liver and Feces

Lipids in liver and feces were extracted according to the method described by Bligh and Dyer [38] using a mixture of methanol and chloroform. The liver and fecal lipid extracts were evaporated to dryness under nitrogen and re-dissolved in isopropanol before quantification of total cholesterol on the Cobas c111 system using the CHOL2 (Cholesterol Gen.2) kit from Roche Diagnostics.

### 4.8. Total Bile Acids in Feces

Total fecal bile acids were measured in freeze-dried feces by the method of Suckling et al. [47], using Chromabond C18 ec (3 mL/200 mg, Macherey-Nagel, Düren, Germany) and Total Bile Acid Assay kit (Diazyme Laboratories, Inc., Poway, CA, USA) on the Cobas c111 system.

### 4.9. Liver Concentrations of 3-Hydroxy-3-Methylglutaryl Coenzyme A (HMG-CoA) Reductase, LDL Receptor, and Protein

HMG-CoA reductase and LDL receptors were measured in liver using the ELISA kits LS-F15758 and LS-F11934 from LifeSpan BioSciences, Inc. (Seattle, WA, USA). Liver protein was quantified with the Bradford dye-binding method [48] using protein assay dye reagent (Bio-Rad Laboratories, Munich, Germany) with bovine serum albumin (Bio-Rad Protein Assay Standard II, Bio-Rad Laboratories, Hercules, CA, USA) as the standard. Concentrations of HMG-CoA reductase and LDL receptors in liver are presented relative to protein.

### 4.10. Statistical Analyses

Statistical analyses were conducted using SPSS Statistics version 23 (SPSS, Inc., IBM Company, Armonk, NY, USA). Independent samples *t*-test was used to compare the BWW group to the control group. All data are presented as the mean ± standard deviation. The cut-off value for statistical significance was set at a probability of 0.05. One rat in the control group was excluded from all statistical analyses due to apparent disease; thus, the results are presented as *N* = 5 for the control group and *N* = 6 for the BWW group.

## 5. Conclusions

Obese Zucker fa/fa rats fed a diet containing water-soluble protein from blue whiting had lower liver and serum cholesterol concentrations and lower concentrations of LDL receptors and HMG-CoA reductase in liver when compared to rats fed a diet with casein as the sole protein source. Concentration of serum total bile acids and fecal excretion of cholesterol and bile acids were similar between the groups. To conclude, the BWW diet led to lower concentrations of serum and liver cholesterol in obese Zucker fa/fa rats, probably due to lower hepatic cholesterol synthesis.

## Figures and Tables

**Table 1:** Contents of indispensable amino acids, the functional amino acid glycine, the conditionally essential amino acid arginine, taurine, and the ratios of lysine/arginine and methionine/glycine, and fatty acids in the diets.

	Control Diet	BWW Diet
Amino acids (g/kg diet)		
Arginine	6.9	7.2
Glycine	3.7	10.0
Histidine	5.6	4.5
Isoleucine	10.2	8.0
Leucine	18.3	15.0
Lysine	16.4	15.0
Methionine	6.9	6.5
Phenylalanine	10.1	8.2
Threonine	8.5	6.6
Valine	13.0	11.0
Taurine	ND	0.2
Lysine/Arginine	2.4	2.1
Methionine/Glycine	1.9	0.7
Fatty acids ^1^ (g/kg diet)		
16:0	6.7	6.7
18:0	2.3	2.3
18:1n-9	12.4	12.6
18:1n-7	0.8	0.8
18:2n-6	29.0	29.8
18:3n-3	3.4	3.5
20:5n-3	ND	0.01
22:5n-3	ND	ND
22:6n-3	ND	0.02

ND: not detected; ^1^ Only fatty acids found in concentrations >0.5 g/kg diet and long-chain n-3 polyunsaturated fatty acids (20:5n-3, 22:5n-3, 22:6n-3) are shown; BWW: blue whiting water-soluble protein.

**Table 2:** Body weight at baseline and at euthanasia, total growth, body weight to square body length ratio, relative liver weight at time of euthanasia, and energy intake at week four.

Parameters	Control Group	BWW Group	*p*-Value
Body weight at baseline (g)	318 ± 8	303 ± 9	0.021
Body weight at time of euthanasia (g)	548 ± 35	510 ± 29	0.077
Growth (% from baseline to endpoint)	72 ± 8	68 ± 7	0.37
Body weight to square body length ratio (kg/m^2^)	10.0 ± 0.6	9.5 ± 0.1	0.10
Relative liver weight (g/kg body weight)	36.5 ± 5.6	32.2 ± 4.8	0.20
Energy intake (kJ/24 h)	456 ± 62	493 ± 27	0.21

Data are presented as the mean ± standard deviation for *N* = 5 rats in the control group and *N* = 6 rats in the BWW group; *p* < 0.05 was considered significant; evaluated by independent samples *t*-test; BWW: blue whiting water-soluble protein.

**Table 3:** Concentrations of serum cholesterols and bile acids.

Biochemical Parameters	Control Group	BWW Group	*p*-Value
Total cholesterol (mmol/L)	5.9 ± 1.0	4.6 ± 0.9	0.039
Cholesteryl ester (mmol/L)	4.6 ± 0.8	3.3 ± 0.7	0.017
LDL cholesterol (mmol/L)	1.5 ± 0.5	0.8 ± 0.3	0.0062
HDL cholesterol (mmol/L)	5.6 ± 0.7	4.1 ± 0.9	0.014
Total bile acids (μmol/L)	16.6 ± 11.8	16.7 ± 9.8	0.99

Data are presented as the mean ± standard deviation for *N* = 5 rats in the control group and *N* = 6 rats in the BWW group; *p* < 0.05 were considered significant; evaluated by independent samples *t*-test; BWW: blue whiting water-soluble protein; LDL: low-density lipoprotein; HDL: high-density lipoprotein.

**Table 4:** Concentration of cholesterol in liver and the fecal excretion of cholesterol and bile acids.

Parameters	Control Group	BWW Group	*p*-Value
Liver cholesterol (µmol/g)	9.4 ± 1.9	6.1 ± 1.6	0.015
Fecal cholesterol (µmol/24 h)	20.8 ± 7.0	16.8 ± 1.7	0.27
Fecal bile acids (µmol/24 h)	5.8 ± 2.6	4.5 ± 1.7	0.36

Data are presented as the mean ± standard deviation for *N* = 5 rats in the control group and *N* = 6 rats in the BWW group; *p* < 0.05 was considered significant; evaluated by independent samples *t*-test; BWW: blue whiting water-soluble protein.

**Table 5:** Composition of the experimental diets.

Contents (g/kg Diet)	Control Diet	BWW Diet
Casein ^1^	216.00	144.00
Blue Whiting Water-Soluble Protein ^2^	-	108.30
Cornstarch	511.67	475.50
Sucrose	90.00	90.00
Cellulose	50.00	50.00
Soybean Oil	70.00	70.00
t-Butylhydroquinone	0.015	0.015
Mineral Mix (AIN-93-MX)	35.00	35.00
Vitamin Mix (AIN-93-VX)	10.00	10.00
l-Methionine	1.60	1.60
l-Cystine	3.00	3.00
Choline Bitartrate ^3^	2.50	2.50
Growth and maintenance supplement (#410751) ^4^	10.00	10.00

^1^ contains 92.5% crude protein; ^2^ contains 61.5% crude protein; ^3^ contains 41% choline; ^4^ contains vitamin B12 (40 mg/kg) and vitamin K1 (25 mg/kg) mixed with sucrose (995 g/kg) and dextrose (5 g/kg); BWW: blue whiting water-soluble protein.

## Supplementary Materials

The following are available online at http://www.mdpi.com/1660-3397/16/5/149/s1, Table S1: Peptide sequences identified in the water-soluble blue whiting protein.
